# Primary ciliogenesis defects are associated with human astrocytoma/glioblastoma cells

**DOI:** 10.1186/1471-2407-9-448

**Published:** 2009-12-17

**Authors:** Joanna J Moser, Marvin J Fritzler, Jerome B Rattner

**Affiliations:** 1 Department of Biochemistry and Molecular Biology, Faculty of Medicine, University of Calgary, Calgary, Alberta, Canada; 2 Department of Cell Biology and Anatomy, Faculty of Medicine, University of Calgary, Calgary, Alberta, Canada

## Abstract

**Background:**

Primary cilia are non-motile sensory cytoplasmic organelles that have been implicated in signal transduction, cell to cell communication, left and right pattern embryonic development, sensation of fluid flow, regulation of calcium levels, mechanosensation, growth factor signaling and cell cycle progression. Defects in the formation and/or function of these structures underlie a variety of human diseases such as Alström, Bardet-Biedl, Joubert, Meckel-Gruber and oral-facial-digital type 1 syndromes. The expression and function of primary cilia in cancer cells has now become a focus of attention but has not been studied in astrocytomas/glioblastomas. To begin to address this issue, we compared the structure and expression of primary cilia in a normal human astrocyte cell line with five human astrocytoma/glioblastoma cell lines.

**Methods:**

Cultured normal human astrocytes and five human astrocytoma/glioblastoma cell lines were examined for primary cilia expression and structure using indirect immunofluorescence and electron microscopy. Monospecific antibodies were used to detect primary cilia and map the relationship between the primary cilia region and sites of endocytosis.

**Results:**

We show that expression of primary cilia in normal astrocytes is cell cycle related and the primary cilium extends through the cell within a unique structure which we show to be a site of endocytosis. Importantly, we document that in each of the five astrocytoma/glioblastoma cell lines fully formed primary cilia are either expressed at a very low level, are completely absent or have aberrant forms, due to incomplete ciliogenesis.

**Conclusions:**

The recent discovery of the importance of primary cilia in a variety of cell functions raises the possibility that this structure may have a role in a variety of cancers. Our finding that the formation of the primary cilium is disrupted in cells derived from astrocytoma/glioblastoma tumors provides the first evidence that altered primary cilium expression and function may be part of some malignant phenotypes. Further, we provide the first evidence that ciliogenesis is not an all or none process; rather defects can arrest this process at various points, particularly at the stage subsequent to basal body association with the plasma membrane.

## Background

Cilia are microtubule-based organelles that extend from the surface of cells and can be classified into two categories, motile cilia with a 9+2 arrangement of microtubules and non-motile (primary) cilia with 9+0 arrangement of microtubules (reviewed in [1]). Most vertebrate cells contain a single non-motile primary cilium that is assembled in a step-wise manner from the distal end of a mature centriole at the centrosome. It is now known that the formation and maintenance of a primary cilium is a complex process involving a wide variety of proteins that include members of the intraflagellar transport (IFT) complex [2-5], pericentrin [6,7], ODF2 [8-10], Cep164 [11], ALMS1 [12,13], EB1 [14] and Cep290 [15-17]. In addition, certain proteins involved in cell cycle progression are linked to primary cilium expression (for review see [18-20]).

Primary cilia have been implicated in signal transduction, cell to cell communication, left and right pattern embryonic development, sensation of fluid flow, regulation of calcium levels, mechanosensation and growth factor signaling. Primary cilia have been detected in the central nervous system (CNS) where the deletion of primary cilia in pro-opiomelanocortin hypothalamic neurons resulted in hyperphagia [2,21]. CNS primary cilia have also been linked to CNS development and the Sonic hedgehog (Shh) signaling pathway [22-25]. Shh signaling components including Patched (Ptc), Smoothened (Smo), Suppressor of fused and Gli transcription factors have been reported to concentrate in CNS primary cilia [26-28]. Astrocytes in the subventricular zone extend a primary cilium into the ventricle suggesting that they may play a role in sensing cerebral spinal fluid (CSF) ion concentration, pH, osmolarity and perhaps changes in protein or glucose levels [29]. Similarly, it is possible that primary cilia in astrocytes may sense levels of neurotransmitters, growth factors, hormones, osmolarity, ions, pH and fluid flow in the extracellular space and relay homeostatic information (or lack thereof) back to the cell body. Diseases associated with faulty primary cilia reinforce the concept that primary cilia are required for the proper development and function of the brain. These diseases include Alström, Bardet-Biedl, Joubert, Meckel-Gruber and oral-facial-digital type 1 syndromes where common neurological related pathologies include obesity, ataxia and mental retardation [30].

The finding that primary cilia are linked to cell cycle regulation and progression has led to suggestions that they may play a role in tumor formation, a supposition that has been validated by several recent studies [31,32]. In the present study, we undertook a comparative investigation of primary cilia in cultured primary human astrocytes and compared them to those found in five human astrocytoma/glioblastoma cell lines. We demonstrate that the primary cilium region in cultured astrocyte cells is structurally complex and includes foci for endocytosis-based signaling. This indicates that there is a spatial link between receptor pathways associated with endocytosis and the primary cilium microenvironment. Importantly, we document that in each of the five astrocytoma/glioblastoma cell lines, fully formed primary cilia are either expressed at a very low level, are completely absent or do not proceed through all the stages of ciliogenesis. In addition, we noted several defects affecting the structure of astrocytoma/glioblastoma centrioles that were not observed in primary human astrocytes. We conclude that aberrant ciliogenesis is a common defect found in cells derived from astrocytomas/glioblastomas and this deficiency likely contributes to the phenotype of these malignant cells.

## Methods

### Antibodies

Primary cilia were marked by one of three separate antibodies: rabbit anti-adenylyl cyclase III (ACIII) antibody (Santa Cruz Biotechnology, Inc., Santa Cruz, CA), rabbit anti-glu tubulin (Chemicon, Temecula, CA), and mouse monoclonal to acetylated tubulin (Sigma-Aldrich, Oakville, ON, Canada). Mouse monoclonals to golgin 97 (CDF4 clone) and TGN38 were from Invitrogen (Burlington, ON, Canada) and Santa Cruz Biotechnology Inc. (Santa Cruz, CA), respectively. Mouse monoclonals to early endosome antigen 1 (EEA1) and lysosomal-associated membrane protein-1 (LAMP-1) were from Abcam Inc. (Cambridge, MA). All antibodies were used according to the manufacturer's specifications.

### Cells

Primary human cerebral cortex astrocyte cells (used between passages 3-5, cat# 1800, ScienCell Research Laboratories, Carlsbad, CA) were cultured in cell specific astrocyte medium containing 2% fetal bovine serum, 1% astrocyte growth supplement and 1% penicillin/streptomycin (ScienCell Research Laboratories). Adherent human U-87 MG (used between passages 14-20, cat# HTB-14, American Type Culture Collection (ATCC), Rockville, MD), T98G (used between passages 10-20, cat# CRL-1690, ATCC), U-251 MG (used between passages 10-20, obtained from Dr. V.W. Yong, University of Calgary, Calgary, AB, Canada), U-373 MG (used between passages 10-20, cat# HTB-17, ATCC), and U-138 MG (used between passages 10-20, cat# HTB-16, ATCC) astrocytoma/glioblastoma cells were cultured in DMEM F12 + 1% L-glutamine (2 mM) (Cambrex, Walkersville, MD) supplemented with 10% fetal calf serum (Gibco, Burlington, ON, Canada), 1% penicillin-streptomycin (Gibco), 1% sodium pyruvate (1 mM) (Gibco) and 1% non-essential amino acids (0.1 mM) (Gibco) at 37°C and 5% CO_2_. Recently the relationship between U-251 MG and U-373 MG cell lines has come into question and analysis has suggested that they likely have a similar origin [33]. Since these two cell lines are still in common use, we have considered them independently in this study but results obtained from these lines are interpreted in light of their apparent relationship.

### Indirect Immunofluorescence (IIF)

Cells were cultured on poly-L-lysine coated coverslips (BD Falcon) for approximately 24 hours at 37°C and then fixed in 100% ice cold methanol for 10 minutes. To minimize non-specific binding of the antibodies, cells were incubated in a blocking buffer containing 10% normal goat serum (Antibodies Incorporated, Davis, CA) and 2% bovine serum albumin (Sigma-Aldrich) for 30 minutes at room temperature (RT). For colocalization studies, cells were incubated with primary antibodies at appropriate working dilutions for 30 minutes at RT. After washing with phosphate buffered saline (PBS), cells were incubated for 30 minutes in a dark chamber with the corresponding secondary goat fluorochrome-conjugated antibodies. Alexa Fluor (AF) 488 (green) or 568 (red) secondary antibodies were from Invitrogen (Burlington, ON, Canada). Subsequently, the slides were washed in several changes of PBS, cell nuclei counterstained with 4',6-diamidino-2-phenylindole (DAPI), mounted in Vectashield (Vector Laboratories, Burlingame, CA) and examined for IIF using a 100× objective on a Leica DMRE microscope equipped with epifluorescence and an Optronics camera. Figures were compiled in Adobe PhotoShop (version 7.0). Appropriate IIF controls with no or only one primary antibody or both secondary antibodies alone or in combination revealed no observable non-specific background staining and no detectable bleed-through between microscope filter sets.

### Electron Microscopy (EM)

Cells were seeded into 35 mm dishes grown to confluence as monolayers over two days, then washed in PBS and fixed in 3% glutaraldehyde in Millonig's phosphate buffer for 1 hour at RT. Post-fixation was in 2% OsO_4 _for 20 minutes. The cells were dehydrated in ethanol, and then infiltrated with Polybed 812 resin (Polysciences). Polymerization was performed at 37°C for 24 hours. Silver-gray sections were cut with an ultramicrotome (Leica) equipped with a diamond knife, stained with uranyl acetate and lead citrate and then examined in a H-700 Hitachi electron microscope. Centriole lengths were obtained by direct measurements of centrioles on electron micrographs and correlated to scale bars automatically generated by the electron microscope on the same electron micrograph.

## Results

To establish a baseline for the evaluation of primary cilia in astrocytoma/glioblastoma cell lines we first investigated the structure and composition of primary cilia in a human primary astrocyte cell line using monospecific antibodies to known primary cilia proteins. These studies were then followed by ultrastructural analysis.

### Human astrocytes express primary cilia that reside within a membrane bound channel herein termed the "cilium-pit" that is also the site of endocytosis

**Figure 1:**
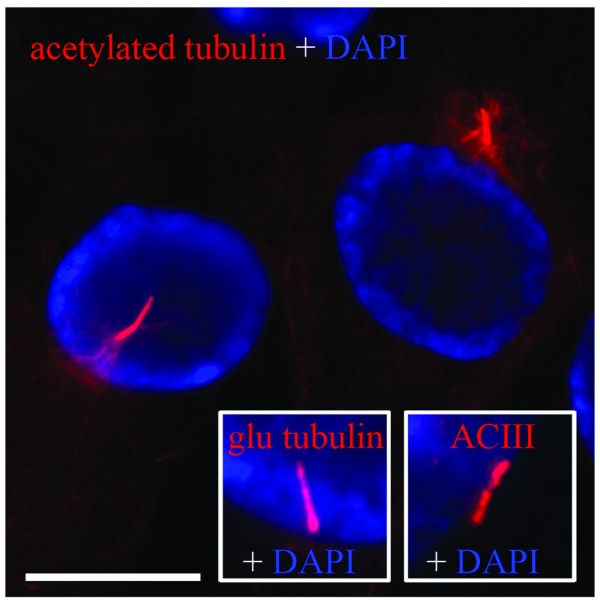
**Normal human astrocytes contain a single primary cilium**. Astrocyte primary cilia were identified extending from the perinuclear region by indirect immunofluorescence (IIF) using antibodies to acetylated (acetyl) tubulin, glu tubulin or adenylyl cyclase III (ACIII) (red) and overlayed with DAPI-stained nuclei (blue). Scale bar = 15 μm.

**Figure 2:**
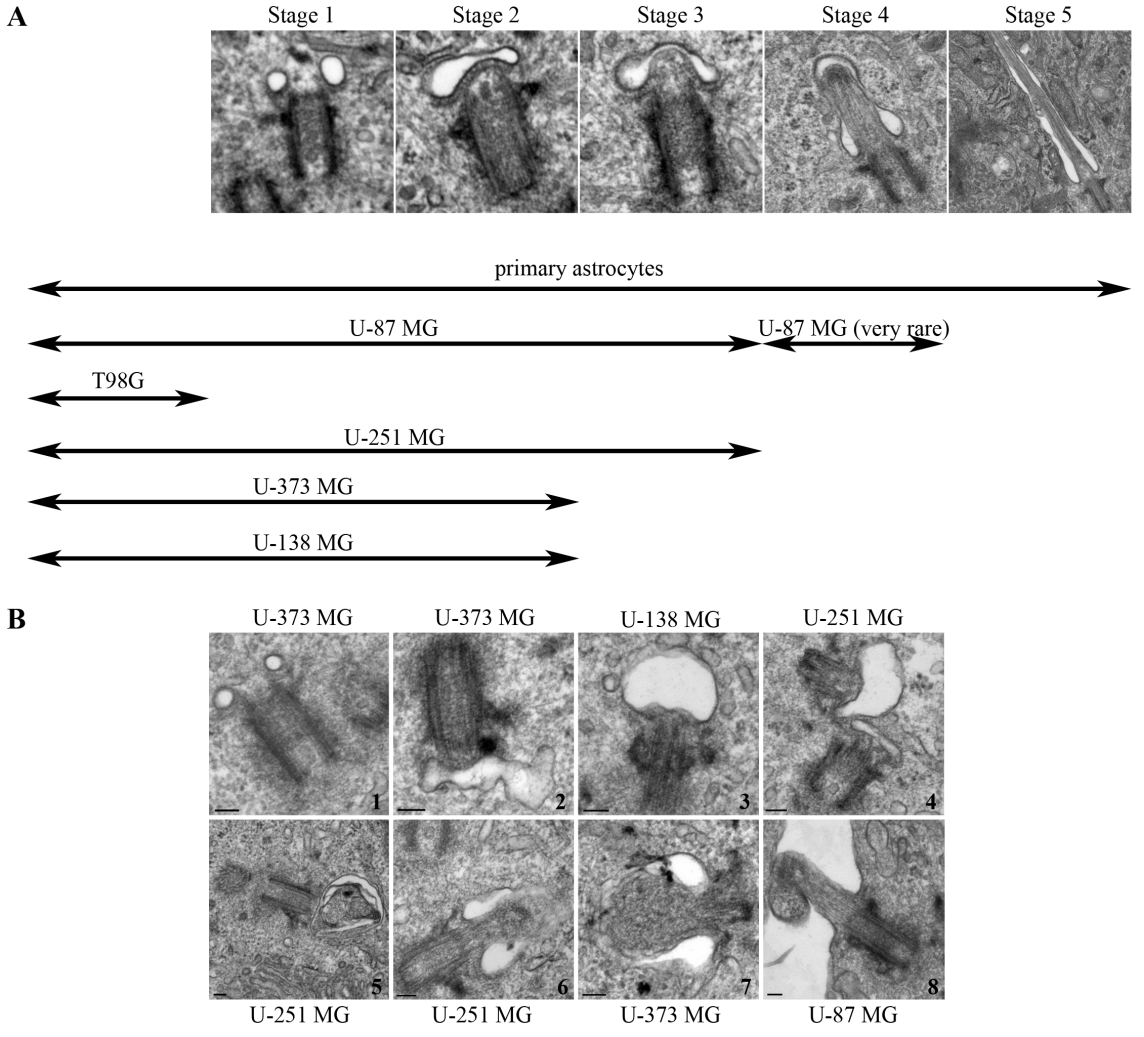
**Astrocyte primary cilia display distinct stages of ciliogenesis**. **(A) **Astrocyte primary cilia ciliogenesis can be divided into five stages as show in the electron micrographs. The double headed arrows indicate that the stages of ciliogenesis displayed by individual astrocytoma/glioblastoma cell lines. At one extreme, T98G glioblastoma/glioblastoma multiforme cells never appear to initiate ciliogenesis, while the other cell lines progress to varying stages. **(B) **Electron micrographs illustrating representative images from four of the cell lines. 1) Early stage 1 common to U-373 MG, 2),3) and 5) are examples of misshaped or swollen vesicles in U-373 MG, U-138 MG and U-251 MG cells, 4) two centrioles in the same cell arrested at stage 2 in U-251 MG cells, 6) and 7) are examples of unusual ciliary buds in U-251 MG and U-373 MG cells, and 8) show an atypical and rare case of an early axoneme in U-87 MG cells.

**Figure 3:**
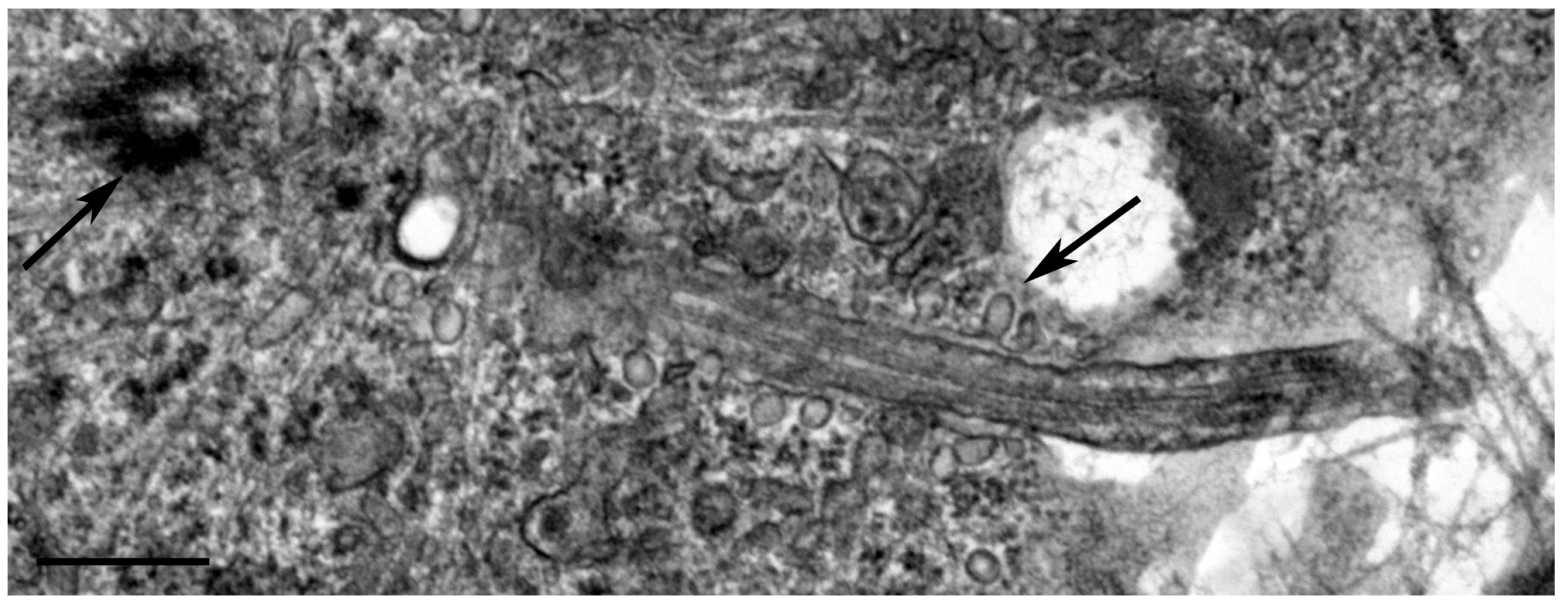
**The human astrocyte primary cilium resides within a membranous invagination the "cilium-pit" that is involved in endocytosis**. Electron micrograph showing a primary cilium extending from the astrocyte cell body into the extracellular matrix. The section was cut obliquely through the basal body (left arrow). The axoneme disappears out of the plane of the section before returning into the focal plane and extends into the extracellular environment. Endocytic vesicles can be seen along the length of the cilium-pit (right arrow, see parallel to shaft of the primary cilium). Scale bar = 250 nm.

Antibodies to acetylated (acetyl) tubulin, glu tubulin (also known as detyrosinated tubulin) and adenylyl cyclase III (ACIII) are markers of primary cilia [34-38] and detected primary cilia in cultured log-phase human astrocytes (Figure 1). A single primary cilium was detected in resting G_0 _phase cells (Figure 1), however astrocytes in G1/S phase did not have detectable primary cilia (data not shown). Examination of astrocytes using electron microscopy revealed cells expressing different stages of ciliogenesis (Figure 2A). Based on our observations and those of previous studies [39-41] we arbitrarily divided the process of ciliogenesis into 5 stages in order to emphasize the early stages of ciliogenesis. Stage 1 was characterized by the presence of two vesicles at the distal end of the basal body; in stage 2 there was a single vesicle associated with the distal end of the basal body and specializations between the vesicle and the distal end of the basal body were prominent; stage 3 was characterized by a ciliary bud that was the first indication of the maturation of the cilium shaft; stage 4 was characterized by the presence of an immature primary cilium with a short axoneme containing characteristic microtubule doublets; finally, at stage 5 a fully formed mature primary cilium was observed (Figure 2A). In cells displaying a mature cilium we noted that the shaft of the primary cilium did not extend completely free of the cell body as described in some cell types (e.g. chondrocytes, renal cells), rather approximately two-thirds of the axoneme was positioned within a membrane bound channel that at its distal end was continuous with the peripheral plasma membrane and was marked by the basal body at its proximal end (Figure 3). This channel can be considered the final expression of the centriole-associated vesicle seen at the early stages of ciliogenesis. We have termed this membrane channel encasing the cilium axoneme the *cilium-pit*.

**Figure 4:**
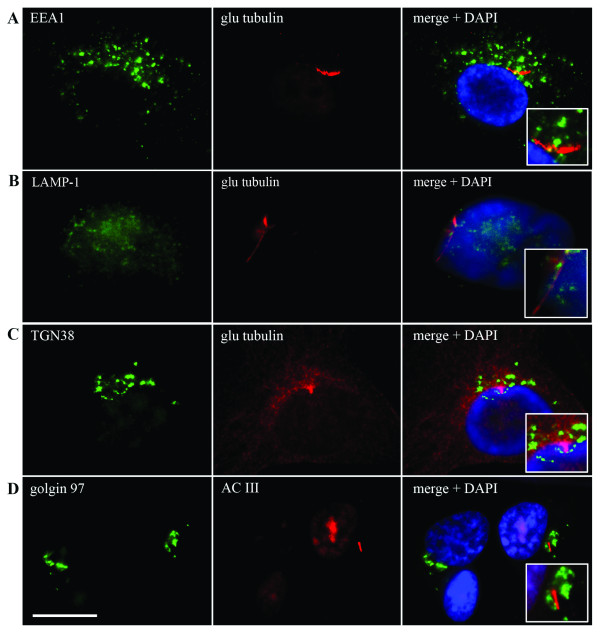
**Endocytosis proteins surround the primary cilium**. Primary cilia in astrocytes were examined by IIF after co-staining with glu tubulin and AC III antibodies (red). The primary cilium was surrounded by organelles involved in endocytosis including **(A) **early endosomes as detected using antibodies directed to early endosome antigen 1 (EEA1), **(B) **late endosomes/lysosomes using antibodies directed to lysosome associated membrane protein 1 (LAMP1), **(C) ***trans *Golgi network using antibodies directed to the *trans *Golgi network 38 (TGN38) and **(D) **the Golgi complex using antibodies directed to golgin 97 (green). Nuclei were counterstained with DAPI. The inset box is an enlargement of the primary cilia in relation to the EEA1, LAMP1, TGN38 and golgin 97 staining. IIF scale bars = 15 μm.

One striking feature of the plasma membrane that lines the pit and lies adjacent to the cell cytoplasm was the presence of what appear to be endocytotic vesicles (Figure 3, right arrow). To verify that the walls of the cilium-pit are sites of endocytosis we stained astrocytes with antibodies to the early endosome component EEA1 (Figure 4A). The early endosome is a major sorting compartment for the endocytic pathway, where many ligands dissociate from their receptors and recycle to the cell surface. Figure 4A illustrates that early endosomes co-localize with the region surrounding the primary cilium. In addition, late endosome structures, such as lysosomes, were also present in this region as shown by staining with antibodies to LAMP-1 (Figure 4B) and antibodies to the *trans *Golgi network (TGN 38) and the Golgi complex (golgin 97) also stained the region bordering the cilium-pit (Figure 4C and 4D respectively). Taken together, we concluded that human astrocytes in culture express primary cilia that are the result of an orderly process of ciliogenesis and that these cilia lay within a specialized structure, the cilium-pit that, in turn, is a site of endocytosis.

Using the aforementioned results as a starting point, the structure and expression of primary cilia was studied in five astrocytoma/glioblastoma cell lines: U-87 MG, T98G, U-251 MG, U-373 MG and U-138 MG using both IIF and electron microscopy.

### Human astrocytoma/glioblastoma cell lines display defective ciliogenesis and centriole structure

**Figure 5:**
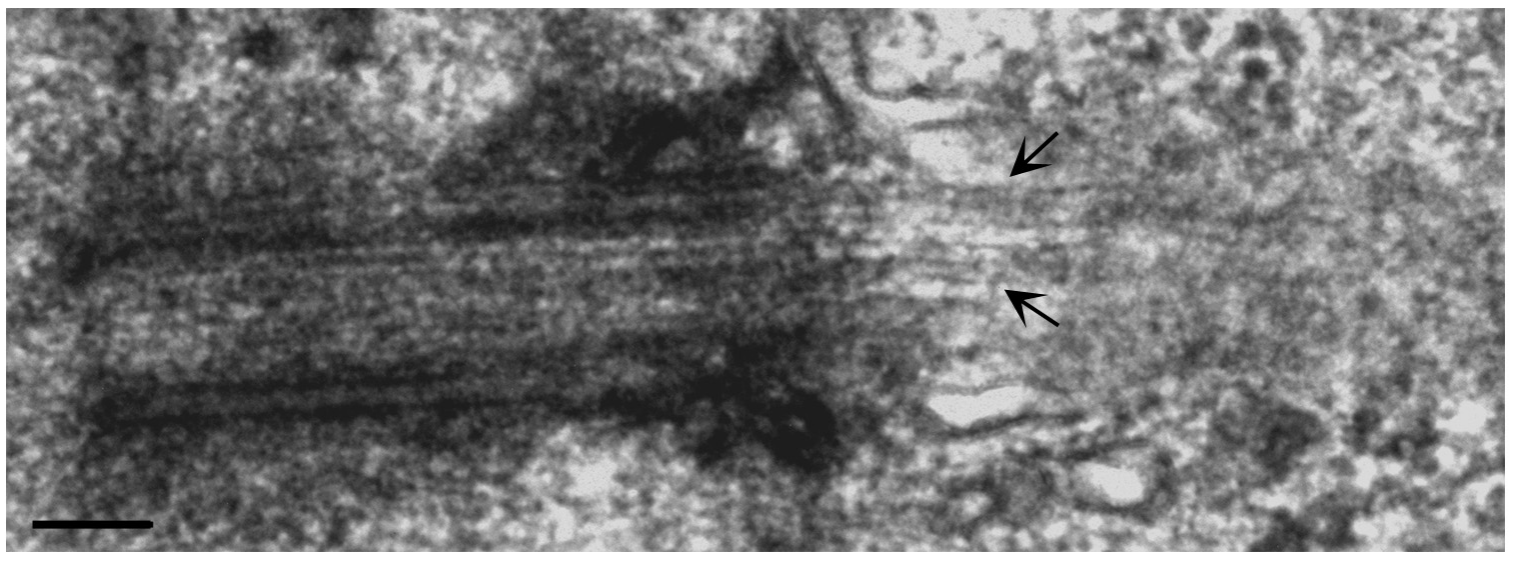
**A primary cilium in the latter stages of ciliogenesis in U-87 MG cells**. Electron micrograph showing well formed microtubule doublets (arrows) with normal spacing between doublets in longitudinal section. Scale bar = 100 nm.

The five astrocytoma/glioblastoma cell lines were cultured in log phase and examined for expression of primary cilia with antibodies to acetyl tubulin, glu tubulin and ACIII as previously described (Results). Cells were scored by two independent observers for the presence of primary cilia over multiple experiments. By IIF, only U-87 MG cells showed any detectable ciliary axonemes and late stage primary cilia were detected in less than 1% of the cells counted (N = 500). To confirm and extend these findings, cells from each astrocytoma/glioblastoma cell line were prepared for electron microscopy and at least 40 cells were examined and scored for both centriole structure and the stages of ciliogenesis. Consistent with our IIF findings, only occasional primary cilia in the latter stages of ciliogenesis were detected by EM of U-87 MG cells (Figure 2B, U-87 MG). The rarity of these immature primary cilia prevented the visualization of cross sections of the axoneme, however in longitudinal sections it was possible to observe well formed microtubule doublets with normal spacing between doublets (Figure 5) suggesting that some aspects of axonemal structure were achieved in these cells. In the remainder of U-87 MG cells, it was common to observe cells with stage 1-3 configurations but later stages were extremely rare (Figure 2A). In contrast, T98G cells did not show any of the various stages of ciliogenesis (Figure 2A). In U-251 MG, U-373 MG and U-138 MG cell lines, cells in stage 1-2 were common but only U-251 MG cells displayed cilia reminiscent of stage 3 (Figure 2A). As previously noted in the Methods section, there are published reports that suggest that U-251 MG and U-373 MG cell lines have a similar origin thus the small differences observed between these two lines in this study may be a result of phenotypic selection while in culture. Although early stages of ciliogenesis were detected in U-87 MG, U-251 MG, U-373 MG and U-138 MG cell lines, various structural abnormalities were common, which included misshapen vesicles and aberrant ciliary buds and axonemes (Figure 2B). Serum starvation is commonly used to arrest cells in G_0 _thereby enhancing the expression of primary cilia. We found that although this was the case in normal primary astrocytes, ciliogenesis was not enhanced by serum starvation in any of the astrocytoma/glioblastoma cell lines (data not shown). In summary, astrocytoma/glioblastoma cell lines displayed a variety of primary cilium defects including: 1) the failure to initiate ciliogenesis, 2) the initiation of ciliogenesis but a failure to proceed past the early stages of the process and 3) progression through ciliogenesis where completion of this organelle is only rarely achieved.

### Abnormalities in centriole structure occur in concert with abnormal ciliogenesis

**Figure 6:**
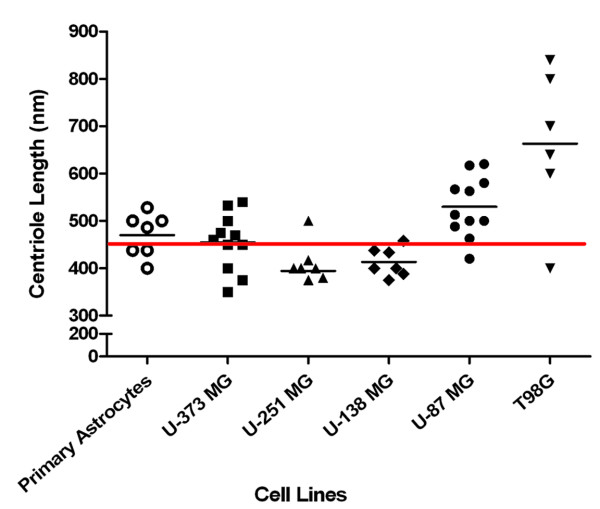
**Comparison of centriole length between human astrocytes and five human astrocytoma/glioblastoma cell lines**. Centrioles were measured directly on electron micrographs and correlated to scale bars automatically generated by the electron microscope on the same micrograph to arrive at centriole length. Individual points indicate single centrioles examined from separate images. The clusters of centrioles from each individual cell line show the variation of centriole lengths from these separate experiments to form a distribution of centriole lengths. Lines in each distribution represent the mean. The average centriole length based on published values is represented by the red line at 450 nm.

In the process of studying the expression of primary cilia in astrocytoma/glioblastoma and documenting the presence of incomplete ciliogenesis a number of additional aberrations associated with cilia-associated structures were noted. Complete longitudinal electron micrograph sections though the centrioles from each of the cell lines included in this study were compared to examine the length of centrioles. In primary human astrocytes, the centriole length ranged between 400-525 nm with a mean length of 470 nm (Figure 6). This was comparable to average centriole lengths of 450 nm reported for a variety of cell types. However, centriole lengths varied among the five astrocytoma/glioblastoma cell lines surveyed (Figure 6). U-373 MG cells had centriole lengths that approximated those found in astrocytes with a distribution range of 370-525 nm and a mean length of 455 nm (Figure 6). U-251 MG and U-138 MG cell lines displayed shorter centrioles (Figure 6). U-251 MG centriole lengths ranged from 375-500 nm with a mean length of 394 nm and U-138 MG centriole lengths ranged from 375-458 nm with a mean length of 413 nm (Figure 6). In contrast, U-87 MG cells contained much longer centrioles with a distribution range of 420-620 nm and a mean length of 530 nm (Figure 6). T98G cells showed the most extreme centriole length with a distribution range of 400-820 nm and a mean length of 663 nm (Figure 6). Since it is difficult to obtain perfect longitudinal sections through centrioles, the number of profiles that qualified for use in Figure 6 are relatively low. Nonetheless, it should be noted that a large number of oblique profiles were observed with dimensions that were consistent with the results shown in Figure 6.

**Figure 7:**
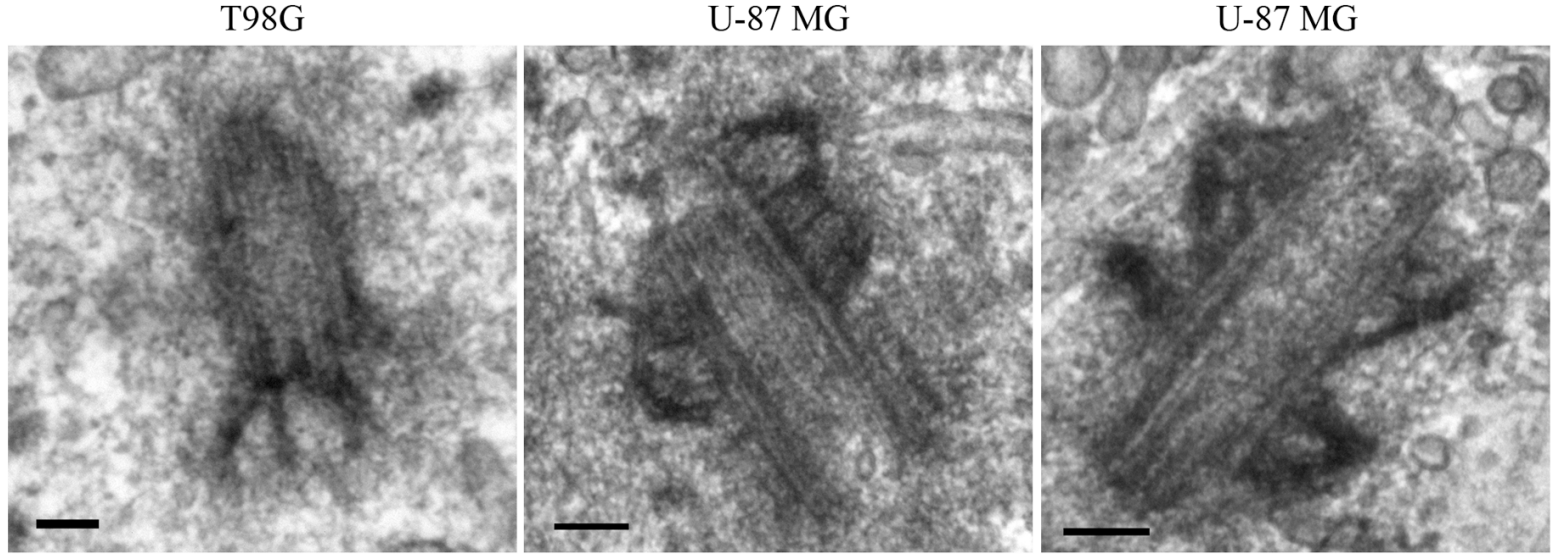
**Centrosome length and centriole appendage architecture is abnormal in T98G and U-87 MG cells**. Electron micrographs showing abnormal distal appendages (left image) and abnormally long centrioles with sub-distal appendage material distributed along their length (centre and right image). Scale bars = 100 nm.

A structural hallmark of mature centrioles was reported to be the presence of two types of appendages termed the distal and sub-distal appendages that extend from the surface of each of the triplet blades of the centriole [42]. Distal appendages are rod shaped structures that are attached to the distal end of the basal body and lie perpendicular to the basal body when examined in longitudinal EM sections. By comparison, sub-distal appendages are triangular shaped structures that are attached to the sub-distal end of the basal body and lie perpendicular to the basal body when examined in longitudinal EM sections [42]. Centrioles displaying these two types of appendages, along with their characteristic morphology, were commonly found in the normal human astrocytes however, abnormal appendage structures in the astrocytoma/glioblastoma cell lines were particularly common in T98G and U-87 MG cell lines (Figure 7). The distal appendages in T98G cells were often orientated parallel to the basal body as opposed to the normal perpendicular direction (Figure 7) and in U-87 MG cells, basal bodies displayed abnormal sub-distal appendages, where the appendages appear to coat the entire length of the basal body (Figure 7). Interestingly, abnormally long centrioles characteristic of U-87 MG and T98G cells also showed abnormal sub-distal and distal appendage structure, suggesting that the length of the centriole may influence the structure of the appendages. Thus, in concert with abnormalities in ciliogenesis, the basal body structure, specifically the length and appendage structure of the basal body, is also perturbed in astrocytoma/glioblastoma cells.

## Discussion

The expression of a primary cilium relies on two events; 1) the activation of ciliogenesis and 2) the orderly progression through the stages of ciliogenesis so that a structurally and functionally competent mature cilium is formed [39-41]. Defects impacting one or both of these events may signal specific disease states. In this study normal primary astrocytes were found to express primary cilia and for the first time it was demonstrated that defects affecting both the activation of ciliogenesis as well as progression through the stages of ciliogenesis occur in cells derived from malignant astrocytomas/glioblastomas. In both cases, this appears to lead to arrest of normal primary cilium expression in these cells. We also found that the progression of ciliogenesis in astrocytoma/glioblastoma cells was unable to be forced or corrected by serum starvation; an observation suggesting that cessation of cell cycling does not affect the ability of astrocytomas/glioblastomas to proceed to later stages of ciliogenesis. In general, the failure to form a functional primary cilium can have at least two major implications: 1) it can alter the cells ability to properly sense and respond to its environment, and 2) it can impact the regulation of cell cycle progression. Thus, the non-communicative and unrestrained growth of astrocytoma/glioblastoma cells may be at least partially defined by defects in the process of ciliogenesis.

Aberrations in ciliogenesis and the resultant absence of mature primary cilia in astrocytoma/glioblastoma cells are not unique to this cell lineage. The absence of primary cilia has been noted in other tumors. For example, ciliogenesis was found to be suppressed in both pancreatic cancer cells and pancreatic intraepithelial neoplastic lesions in human pancreatic ductal adenocarcinoma (PDAC) as well as in three separate mouse models of PDAC driven by an endogenous oncogenic Kras allele [43]. Interestingly, inhibition of the Kras effector pathway restored ciliogenesis suggesting that ciliogenesis in these cells may be actively repressed by oncogenic Kras. Unfortunately, the ultrastructure of these cells was not reported making it difficult to determine if these cells failed to initiate ciliogenesis or that they did so but then failed to proceed through ciliogenesis as observed in the astrocytomas/glioblastomas of our study.

Following the completion of our study and the submission of this manuscript two important studies have been published which directly relate to our findings [31,32]. In the first study, the authors present evidence showing that primary cilia play a role in basal cell carcinoma (BCC). Specifically, ciliary ablation strongly inhibited BCC-like tumors induced by an activated form of Smo while removal of cilia accelerated tumors induced by activated Gli2, a transcriptional effector of Hedgehog (Hh) signaling. Similarly and directly related to our study, the study by Han *et al*. (2009) showed that genetic ablation of cilia is able to block medulloblastoma formation when the tumor was driven by a constitutively active Smo protein while removal of a cilium was required for medulloblastoma growth by a constitutively active Gli2 [32]. Further, it was found that the presence or absence of cilia was associated with specific variants of human medulloblastoma primary cilia and were found in medulloblastomas with active Hh or WNT signaling but not in most medulloblastomas in other distinct molecular subgroups. Taken together, both of these studies demonstrate that primary cilia function as unique signaling organelles that can either mediate or suppress tumorigenesis depending on the nature of the oncogenic initiating event. Interestingly, Katayma *et al*. (2002) reported that Ptc and Smo mRNA expression in human astrocytic tumors are inversely correlated with malignancy histology and this relationship also applied to tumor-derived cell lines [44]. This study also indicated that mRNA levels for Ptc and Smo proteins were weakly expressed in three of the five cell lines included in our study (U-87 MG, U-251 MG and T98G). Taken in context with our results, it would appear that the complex relationship of primary cilia, the Hh signaling pathway and tumorigenesis is likely a feature of astrocytoma cells. Importantly, our data expands previous studies by indicating that ciliogenesis is not an all or none processes but rather can be truncated at different stages based on underlying molecular defects. In the future, it will be important to determine to what degree specific defects in ciliogenesis progression impact tumorigenesis.

In addition to identifying ciliogenesis defects within astrocytoma/glioblastoma cell lines, our study also demonstrated the presence of structural aberrations in the centrioles of some astrocytoma/glioblastoma cell lines. Interestingly, Lingle and Salisbury (1999) reported unusually long centrioles similar to those reported here, as well as disruptions in the 'cartwheel' structure of the centriole in human breast cancer tissue [45]. A recent report by Keller *et al*. (2009) has implicated the protein POC1 in the control of centiolar length [46]. POC1 protein abnormalities may be a candidate in future studies of astrocytomas, glioblastomas or other cancer cells to determine if this protein is non-functional in cancer cells. Another recent study has shown that two centriolar proteins CPAP and CP110 play antagonizing roles in controlling the length of centrioles where overexpression of CPAP leads to strikingly long centrioles [47]. Although it is unclear if ciliogenesis defects and centriole structural defects are related, it is interesting to note that unusual centriole length was correlated with aberrant distal and sub-distal appendages characteristic of T98G and U-87 MG cell lines (Figures 6 and 7). Our results suggest that alterations in centriole length may prevent the formation of properly structured appendages that are critical for basal body attachment to the plasma membrane. It has been reported that a lack of distal and sub-distal appendages in Odf2 (-/-) cells prevents docking of the mother centriole to the plasma membrane thus inhibiting ciliogenesis [8]. Failure to attach properly to the plasma membrane may be related to defects of ciliogenesis and it is possible that the same defect(s) that forms the basis for aberrant ciliogenesis also impacts centriole structure.

A review of the literature reveals that primary cilia are either positioned at the surface of the cell so that the entire axoneme extends outward completely free of the cell body [48-50] or in a channel continuous with the plasma membrane so that only one third of the cilium extends outward from the cell surface [50-53]. Each configuration appears to be cell type specific and our study clearly shows that the internal configuration is characteristic of primary astrocytes. We also found this configuration in U-87 MG cells that only on rare instances displayed the latter stages of ciliogenesis.

To our knowledge, this is the first report to show that the astrocyte cilium-associated channel, which we have termed the cilium-pit, is a site of endocytosis. The association of the primary cilium with the cilium-pit may serve several functions. First, the pit may function to concentrate proteins, ions, hormones or neurotransmitters so that they can better exert their effects on the membranes of either the cilium-pit or the primary cilium. Second, the pit may allow endocytotic associated signaling pathways to be linked and/or coordinated with signaling pathways associated with the primary cilia. Thus, the formation of both a mature cilium and a cilium-pit in certain cell types may be crucial to essential cell signaling.

Recently Kovacs *et al*. (2008) reported that beta-arrestins are required to mediate the activity-dependent interaction of Smo and the kinesin motor protein Kif3A in the primary cilium [54]. They showed that beta-arrestins are required for endocytosis of Smo and signaling to Gli transcription factors to mediate the effects of Hh on developmental processes which, if deregulated, may lead to tumorigenesis [54]. When beta-arrestins were depleted using siRNA, Smo was no longer localized to the primary cilia and Gli was not activated [54], which suggested that endocytosis may be a key intracellular transport mechanism for the subcellular localization of signaling proteins. In future it will be important identify exactly which endocytic linked pathways function in the cilium-pit region.

It should be noted that the detection of a cilium-pit in astrocytes is reminiscent of the flagellar pocket found in single celled trypanosomes [55,56]. Like the cilium-pit, the flagellar pocket is an invagination of the plasma membrane that forms a structure that encases the proximal end of a cilium and is an active site of secretion and endocytosis. However, the flagellar pocket differs from the cilium-pit in several ways. First, the flagellar pocket is associated with a motile rather than a non-motile cilium and the pocket is much shorter in length relative to the cilium. Second, the flagellar pocket has a highly structured neck region that is not found in the cilium-pit. Since the flagellar pocket is a hallmark of trypanosomes and has an organization that distinguishes it from the cilium-pit, we suggest that, in order to avoid confusion, it is appropriate to avoid using the flagellar pocket term to denote the region around the non-motile primary cilium found in human astrocytes.

## Conclusions

The major finding of this report is that primary cilia are expressed by human astrocytes but this expression is disrupted generally during early ciliogenesis in cultured astrocytoma/glioblastoma cells. This finding is important for several reasons. First, it indicates that defects in primary cilium ciliogenesis do occur in astrocytomas and glioblastomas thus providing an impetus for further studies of the relationship between primary cilium defects and this type of malignancy. Second, it highlights the early events of ciliogenesis as a critical time in the ciliogenesis process and emphasizes the importance in not only identifying proteins that are critical to this period but investigating which of these proteins might be correlated with not only cancer but other known ciliopathies. Finally, our results show that primary cilia in astrocytes are adjacent to endosomal markers. Thus, the disruption in ciliogenesis also disrupts the formation of a site of endocytosis-based signaling.

## Competing interests

The authors declare that they have no competing interests.

## Authors' contributions

JJM carried out cell culturing for all experiments, performed the indirect immunofluorescence assays and wrote the first draft of the manuscript. JBR carried out the electron microscopy studies. All authors conceived the study, participated in the design and interpretation of the data, read and approved the final manuscript.

## Pre-publication history

The pre-publication history for this paper can be accessed here:

http://www.biomedcentral.com/1471-2407/9/448/prepub
